# A Comparative Evaluation of Digital Radiography and Ultrasound Imaging to Detect Periapical Lesions in the Oral Cavity

**DOI:** 10.7759/cureus.30070

**Published:** 2022-10-08

**Authors:** Surendra Jaswal, NeelKant Patil, Mohit P Singh, Ashish Dadarwal, Vineet Sharma, Akhil K Sharma

**Affiliations:** 1 Oral Medicine and Radiology, Pacific Dental College and Hospital, Udaipur, IND; 2 Oral and Maxillofacial Radiology, Rajasthan Dental College and Hospital, Jaipur, IND; 3 Prosthodontics, Rajasthan University of Health Sciences (RUHS) College of Dental Sciences, Jaipur, IND; 4 Oral Medicine and Radiology, Pearl Dental and Orthodontic Avenue, Jaipur, IND

**Keywords:** periapical cyst, periapical granuloma, periapical abscess, ultrasound imaging, digital radiography

## Abstract

Purpose

This study evaluates the efficacy of digital radiography and ultrasound (USG) for the distinction between periapical cysts and granulomas, determines the nature and extent of the periapical lesion, visualizes the lumen of the lesion, assesses its size, content, and vascularity.

Material and Methods

Thirty patients, ages 18 to 40, with well-defined periapical radiolucencies on maxillary or mandibular teeth, indicated for the extraction or periapical surgery, underwent digital radiography examination using the paralleling technique, followed by USG examination. A sonologist evaluated the lesions' size, echogenicity, and vascular content. The diagnosis was compared to histopathological examinations of tissues obtained through extraction or periapical surgery.

Results

The diagnostic value of USG compared to the histopathological diagnosis of the periapical cyst was greater than that of the radiographic diagnosis, with an ultrasonographic diagnostic sensitivity (SN) value of 60% and a radiographic diagnostic SN value of 40%, respectively. The diagnostic value of USG imaging against the histopathological diagnosis of periapical granuloma was slightly lower than that of digital radiography, with an SN value of 72.2% for USG and 83.33% for digital radiography. However, the specificity (SP) value and precision of USG imaging were superior to those of digital radiographic diagnosis. USG imaging and radiographic diagnosis had 58.33% and 50% SP values, respectively. In cases of periapical abscess, the diagnostic values of USG against histopathological diagnosis were lower than those of radiographic diagnosis, which had an SN value of 100%.

Conclusion

USG with color doppler is a more effective tool than digital radiography for diagnosing periapical lesions. The echo structure of the lesions and the presence of vascularity on USG with color doppler correlated with histopathology better than the radiological diagnosis.

## Introduction

In most cases, periapical lesions are diagnosed and treated based on radiographic findings. After dental caries or trauma, the most common periapical radiolucencies are periapical granulomas, cysts, and periapical abscesses [1,2]. Periapical lesions are typically diagnosed, treated, and monitored using conventional radiography. A significant reduction in radiation exposure of 50-80% has made digital radiography a popular alternative to conventional radiography in the past two decades [3]. A digital radiograph replaces an x-ray film with an image displayed on a computer using an electronic image receptor. As compared with conventional radiography devices, digital radiography has the advantage of almost instantaneous image availability after exposure. As a result, multiple angles can be taken to locate canals, determine root curvatures, verify working lengths, and intermediate obturation. In recent studies, however, digital radiography has not proven more accurate in diagnosing periapical lesions than conventional radiography, even after image processing and enhancement [4-6]. To overcome these shortcomings, new and more promising methods must be evaluated.

Using ultrasound (USG) imaging to diagnose periapical lesions is a simple and reproducible technique that can supplement conventional and digital radiography. The mechanism of action of USG for periapical lesions is based on ultrasonic waves generated by the electrical stimulation of a piezoelectric crystal called a transducer. The beam hits an interface between tissues of varying acoustic impedance, causing some of the sound waves to reflect to the transducer. The echoes are then converted into electrical impulses that can be viewed on an oscilloscope, giving a picture of the tissues. USG, in conjunction with color doppler, depicts the content and vascularization of the lesions, which is critical in detecting periapical lesions and distinguishing periapical cysts from granulomas [7-9]. Therefore, USG imaging can complement conventional and digital radiography, as it is less costly and less hazardous concerning radiation exposure than computerized tomography [10]. Due to the limitations of routinely used digital radiography, correct diagnoses of periapical lesions can assist in predicting treatment outcomes and decrease root canal treatment failures associated with improper diagnosis [8].

Therefore, the present study was undertaken to determine the exact nature and extent of the periapical lesion, visualize the contents of the lumen of the periapical lesion, assess the size, content, and vascularity of the periapical lesion using USG, and evaluate the efficacy of digital radiography and USG in differentiating periapical cysts from granulomas.

## Materials and methods

This study was conducted in the department of oral medicine and radiology of Rajasthan Dental College and Hospital in Jaipur for one year. The study examined 30 patients between 18 and 40 years with well-defined periapical radiolucency involving either maxillary or mandibular teeth and indicated extraction or periapical surgery by digital radiography using paralleling technique and USG. Lesions associated with systemic conditions (such as hyperparathyroidism, Paget's disease, fibrous dysplasia, multiple myeloma, osteoporosis), endo-perio lesions, or ill-defined radiolucency, and endodontically or orthodontically treated patients were excluded.

A study approval was obtained from the Institutional Ethical Committee of Rajasthan Dental College and Hospital, Jaipur. Based upon inclusion and exclusion criteria, 30 subjects became part of this study and were assessed using a pre-designed and structured methodology.

The patients underwent digital radiography using RadioVisioGraphy (RVG). The digital images were observed, and the lesion size was measured in supero-inferior and mesio-distal directions using a scale installed in RVG (SOPIX inside system; Acteon, Gandhinagar, Gujarat) software. A paralleling technique was used to take preoperative periapical radiographs. Periapical granulomas were diagnosed as well-circumscribed periapical radiolucency measuring less than 1.5 cm in diameter, periapical cysts were characterized by well-defined periapical radiolucency with sclerotic borders measuring greater than 1.5 cm, and periapical abscesses were characterized by ill-defined periapical radiolucency with diffuse margins.

All the subjects underwent ultrasonographic examination using a diagnostic USG system (Prosound α6; Hitachi Akola Medical Ltd., Tokyo, Japan) with a multi-frequency linear transducer using 7-11 MHz frequency at a private diagnostic center in Jaipur. A sonologist examined the lesions for their size, echogenicity, and vascular content. The periapical abscesses were evaluated as hypoechoic, ill-defined, and peripherally vascularized lesions, while the periapical granulomas showed a poorly defined hypoechoic area with abundant vascular supply, whereas the periapical cysts showed anechoic well-contoured cavities without vascularization, surrounded by reinforced bone walls.

**Figure 1:**
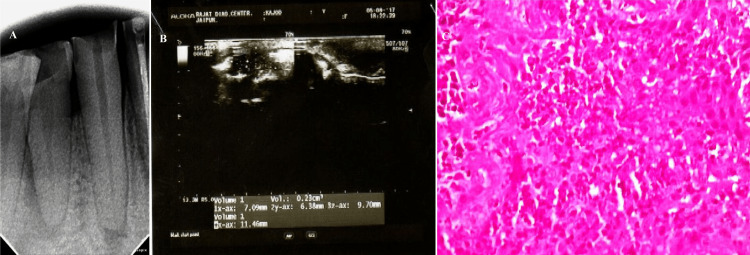
(A) Digital radiograph image showing ill-defined periapical radiolucency in relation to 42, suggestive of periapical abscess; (B) USG image revealing a hypoechoic ill-defined and peripherally vascularized lesion; (C) photomicrograph showing periapical abscess with inflammatory cells 42 = mandibular right lateral incisor, USG = ultrasound

**Figure 2:**
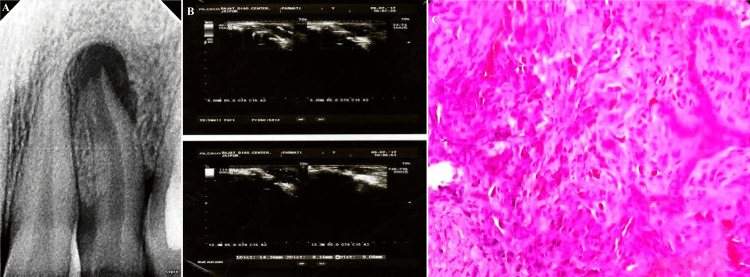
(A) Digital radiographic image showing well-circumscribed periapical radiolucency measuring less than 1.5 cm, suggestive of periapical granuloma in relation to 22; (B) USG image showing rich vascularity on color Doppler examination suggestive of periapical granuloma; (C) photomicrograph of periapical granuloma with granulomatous tissue 22 = maxillary left lateral incisor, USG = ultrasound

**Figure 3:**
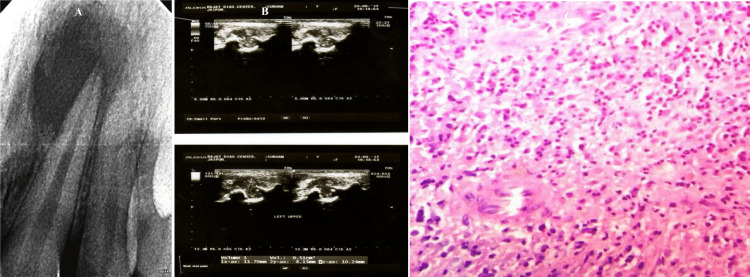
(A) Digital radiograph image showing well-circumscribed periapical radiolucency in relation to 21, 22 measuring more than 1.5 cm, suggestive of periapical cyst; (B) USG images reveal an anechoic well-contoured cavity surrounded by reinforced bone walls without vascularization; (C) photomicrograph showing periapical cyst lined with stratified squamous epithelium 21 = maxillary left central incisor, 22 = maxillary left lateral incisor, USG = ultrasound

By using digital radiography and USG, periapical abscesses, cysts, and granulomas were diagnosed; peri radicular surgery was performed, and periapical biopsies were obtained. The surgical specimens were processed for routine histopathological examination after fixing in 10% buffered formalin. Histopathological evaluation of tissues acquired by extraction or periapical surgery was used to compare the diagnosis (Figure 1-3).

All the study participants have been explained of the need and design of the study. The potential benefits of undergoing thorough clinical, radiographic, and USG investigations were also presented to all patients. After obtaining informed consent, the individuals were included in the study.

The present study uses statistical software (SPSS version 23; IBM Inc., Armonk, New York) to perform descriptive and inferential statistical analysis. The qualitative data were expressed in proportion and percentages, odds ratio with a 95% confidence interval, and the quantitative data expressed as mean and standard deviations. The difference in proportion was analyzed by using the chi-square test. The difference in means among the groups was analyzed using the student T-test for parametric data. The various imaging modalities' diagnostic accuracy, sensitivity, and specificity were calculated.

## Results

The 30 subjects were divided into five age groups with an interval range of five years, except for the first age group of three years (i.e., 18-20 years). There were nine (30%) subjects between the ages of 18-20 years, followed by seven (23.33%) subjects between the ages of 35-40. Out of 30 subjects, 20 (66.67%) were females, and 10 (33.33%) were males; six (20%) showed lesions in the mandible, whereas 24 (80%) showed lesions in the maxilla (Table 1).

**Table 1:** Characteristics of the study population

Characteristics	Group	Number	Percentage
Age group	<20	9	30
	21 to 25	4	13.33
	26 to 30	6	20
	31 to 35	4	13.33
	>35	7	23.33
Sex	Female	20	66.67
	Male	10	33.33
Site	Mandible	6	20
	Maxilla	24	80

Out of 30 cases of periapical pathology, a periapical abscess was seen in two (6.67%), periapical granuloma in 21 (70%), and periapical cyst in seven (23.3%), respectively. Two cases of periapical abscess showed a mean superior-inferior length of 6.0 mm and a mean mesiodistal length of 6.50 mm. In 21 cases of periapical granuloma, the mean superior-inferior length was 11 mm, and the mean mesiodistal length was 9.47 mm. In seven cases of the periapical cyst, the mean superior-inferior length was 16.29 mm, and the mean mesiodistal length was 15 mm (Table 2). USG showed the lesion within the bone in three dimensions and their content by measuring the density values (in terms of fluids or tissues) and blood vessels using a color power doppler. Based on the ultrasonographic characteristics, the periapical lesions were grouped into the periapical cyst, periapical granuloma, and periapical abscess, depending upon the contents being cystic, solid, or both).

**Table 2:** Distribution of the cases according to radiographic diagnosis PA = peri-apical

Measurement	Periapical lesion	Number	Mean	Standard deviation	p-value
Superio-inferior	PA abscess	2	6.00 mm	1.41 mm	<0.001
	PA cyst	7	16.29 mm	3.20 mm	
	PA granuloma	21	9.71 mm	2.95 mm	
	Total	30	11.00 mm	4.23 mm	
Mesio-distal	PA abscess	2	6.50 mm	3.54 mm	<0.001
	PA cyst	7	15.00 mm	3.56 mm	
	PA granuloma	21	7.90 mm	2.43 mm	
	Total	30	9.47 mm	4.11 mm	

The ultrasonographic study showed the features of periapical lesions in all 30 cases, which enabled categorization into groups. One (3.33%) subject was diagnosed with a periapical abscess with the following characteristics: the maximum diameter of the periapical abscess was 7.00 mm (mean) with a standard deviation of 1.41 mm and a significant p-value of 0.018, hypoechoic echogenicity with scattered dense internal echoes, mildly irregular walls (ill-defined walls), and no internal vascularization. Eleven (36.67%) subjects were diagnosed with the periapical cyst, which showed the following characteristics: the maximum diameter of the periapical cyst was 11.10 mm (mean) with a standard deviation of 3.57 and a significant value of 0.018, anechoic echogenicity, well-defined margins with smooth contours, and no evidence of internal vascularization on the application of color power doppler. Eighteen (60%) subjects were diagnosed with periapical granuloma, which showed the following characteristics: the maximum diameter of periapical granuloma was 8.56 mm (mean), with a standard deviation of 1.48 and a significant p-value of 0.018, hypoechoic echogenicity, ill-defined margins (mildly irregular walls), and rich internal and peripheral vascularization on the application of color power doppler (Table 3,4).

**Table 3:** Ultrasonographic diagnosis PA = peri-apical

Diagnosis	Diagnosis criteria	Number	Percentage
Ultrasonographic diagnosis	PA abscess	1	3.33
	PA cyst	11	36.67
	PA granuloma	18	60
Echogenicity	Anechoic	11	36.67
	Hypoechoic	19	63.33
Margins	Ill-defined	18	60
	Well-defined	12	40
Vascularization	Absent	11	36.67
	Peripherally	19	63.33

**Table 4:** USG maximum diameter of the lesion of the histopathological diagnosis PA = peri-apical, USG = ultrasound

Histopathological diagnosis	Number	Mean ± standard deviation	p-value
PA abscess	2	7.00±1.41 mm	0.018
PA cyst	10	11.10±3.57 mm	
PA granuloma	18	8.56±1.48 mm	

According to histopathological diagnosis, out of 30 subjects, two (6.67%) had periapical abscesses, 10 (33.33%) had periapical cysts, and 18 (60%) subjects had periapical granuloma. According to the lesion content, out of 30 cases, two (6.67%) cases showed inflammatory cells as the chief content. They were diagnosed as periapical abscesses, composed chiefly of a central area of disintegrating polymorphonuclear leukocytes, surrounded by viable leukocytes, occasionally lymphocytes, cellular debris, necrotic materials, and bacterial colonies. Eighteen (60%) cases showed granulation tissue as their chief content and were diagnosed as periapical granuloma histopathologically. Ten (33.33%) cases had a fluid cavity as the primary content and were diagnosed as periapical cysts. The fluid cavity was lined by stratified squamous epithelium. The epithelium-lined fluid cavity (lumen) contained watery straw-colored and blood-tinged fluid to semisolid materials, with a low protein concentration that stains palely eosinophilic. Other contents were cholesterol, keratin, hyaline or Rushton body, bundles of collagen fibers, fibroblasts, small blood vessels, lymphocytes, plasma cells, polymorphonuclear leukocytes, and multinucleated giant cells.

The diagnostic values of USG and radiographic diagnosis versus histopathological diagnosis

Out of 30 cases, USG diagnosed 20 (66.67%) cases, and 10 (33.33%) cases were not diagnosed against histopathological diagnosis. In contrast, digital radiography diagnosed 21 (70%) cases, and only nine (30%) cases were not diagnosed against histopathological diagnosis. The diagnostic values of the radiographic diagnosis against the histopathological diagnosis of periapical granuloma with a sensitivity (SN) value of 83.33%, specificity (SP) value of 50%, positive predictive value (PPV) of 71.43%, negative predictive value (NPV) of 66.67%, and an accuracy of 70%. The diagnostic values of the USG diagnosis against the histopathological diagnosis of periapical granuloma with an SN value of 72.22%, SP value of 58.33%, PPV of 72.22%, NPV of 58.33%, and accuracy of 66.67%.

The diagnostic values of the radiographic diagnosis against the histopathological diagnosis of the periapical cyst with an SN value of 40%, SP value of 85%, PPV of 57.14%, NPV of 73.91%, and an accuracy of 70%. The diagnostic values of the USG diagnosis against the histopathological diagnosis of the periapical cyst with an SN value of 60%, SP value of 75%, PPV of 54.55%, NPV of 78.95%, and an accuracy of 70%. The diagnostic values of the radiographic diagnosis against the histopathological diagnosis of periapical abscess with an SN value of 100%, SP value of 100%, PPV of 100%, NPV of 100%, and an accuracy of 100%. The diagnostic values of the USG diagnosis against the histopathological diagnosis of periapical abscess with an SN value of 50%, SP value of 100%, PPV of 100%, NPV of 96.55 %, and an accuracy of 96.67% (Table 5).

**Table 5:** Diagnostic values of the radiographic diagnosis and USG against the histopathological diagnosis of PA granuloma, PA cyst, and PA abscess PA = peri-apical, USG = ultrasound

Value (in %)	Histopathological diagnosis					
	PA granuloma		PA cyst		PA abscess	
	Radio	USG	Radio	USG	Radio	USG.
Sensitivity	83.33	72.22	40	60	100	50
Specificity	50	58.33	85	75	100	100
Positive predictive value	71.43	72.22	57.14	54.55	100	100
Negative predictive value	66.67	58.33	73.91	78.95	100	96.55
Accuracy	70	66.67	70	70	100	96.67

## Discussion

Dental radiography (intraoral periapical radiograph) is essential in diagnosing periapical pathologies. Intraoral periapical radiographs help diagnose, plan, and monitor treatment outcomes during follow-up [7].

In our study, the radiographs and USG showed the presence of periapical pathology in all cases. The USG measurements were smaller than corresponding radiographic measurements in periapical cysts and periapical granuloma cases. On USG examination, the mean maximum diameter in the case of the cyst was 11.10 mm, and in the case of periapical granuloma, it was 8.56 mm. On radiographic examination, the mean maximum diameter in the cyst case was 16.29 mm, and in periapical granuloma, it was found to be 9.71 mm (Table 2,4). These findings were in accordance with Gunddapa et al. and Raghav et al. [7,11,12]. However, a few others did not measure the dimensions but only considered the diagnosis [10,13]. The size variations in USG lesions could be attributed to the acoustic shadow cast by the edges of the bony cavity, which depicts smaller hypoechoic or anechoic lesions [7].

In the present study, out of 10 cases of periapical cysts diagnosed histopathologically, six cases were identified as periapical cysts by USG, and only four were diagnosed by digital radiography. The above observations suggest that the diagnostic value of USG against the histopathological diagnosis of the periapical cyst was higher than that of the radiographic diagnosis, with an ultrasonographic diagnostic SN value of 60% and a radiographic SN value of 40%, respectively (Table 5). These results showed that USG has a higher diagnostic value than digital radiography.

USG accurately identifies the content of periapical lesions and the presence or absence of vascularization. These results confirm that USG imaging is an advantageous technique that can give important diagnostic information regarding periapical lesions. These findings were in accordance with previous USG imaging studies that reported accurate periapical lesion identification [14].

The diagnostic value of USG imaging against the histopathological diagnosis of periapical granuloma was slightly less than the diagnostic value of the radiograph, with an SN value of 72.2% in USG and 83.33% in digital radiography. However, the SP value and accuracy of USG imaging were greater than digital radiographic diagnosis. The SP value of USG imaging and the radiographic diagnosis was 58.33% and 50%, respectively (Table 5).

In cases of periapical abscess, the diagnostic values of USG against histopathological diagnosis were lower, with an SN value of 50%, compared to the diagnostic values of radiographic diagnosis, which had an SN value of 100% (Table 5). The above result can be explained by the fact that our study only found two cases of periapical abscesses and that USG imaging required a skilled operator and thin labial or buccal cortical plates to detect periapical lesions [15]. USG imaging provides valuable diagnostic information about anterior periapical lesions, as demonstrated in this study. According to these findings, an oral physician skilled in USG can diagnose periapical lesions without periapical radiographs if only clinical findings are available.

USG real-time imaging is easily reproducible and convenient to use. In comparison to other advanced imaging modalities, the equipment is relatively inexpensive. Once the observer has been trained, the images obtained are simple to read. They are easy to save and retrieve. A working diagnosis could be made quickly by obtaining a real-time image, potentially avoiding unnecessary ionizing radiation exposure for the patient. There are no negative effects of USG waves in the tissues due to the USG examination. Even if the effect of frequent USG examinations is unknown, the risk of taking radiographs is undeniably higher [16-18]. This study established real-time USG imaging as a reliable diagnostic technique for distinguishing periapical lesions, such as periapical cysts and granulomas, based on the echo texture of their contents and the presence of vascularity using color doppler in the anterior part of the mouth where the buccal cortical bone is thinned. USG real-time imaging is reproducible and straightforward to use.

The limitations of USG imaging include operator dependence and the requirement of a thin buccal cortical plate for USG waves to penetrate and diagnose periapical lesions. By using digital radiography, periapical diseases can be detected, but their nature cannot be determined. USG imaging underestimates the size of the lesions while giving accurate information about the disease's pathology [15,18].

## Conclusions

The present study confirms that ultrasound imaging is a reliable diagnostic technique for differentiating periapical lesions, unlike digital radiography, and provides sufficient information regarding the nature of the periapical lesions. A USG diagnosis matched the echo structure of the lesions and the presence of vasculature better than a radiological diagnosis when compared with a histopathological diagnosis. Nevertheless, none of the imaging techniques are superior to the others, as USG underestimates the size of lesions but can provide accurate information about their pathological nature, whereas digital radiography can detect periapical disease but not it's content. So, USG imaging can be used in conjunction with conventional and digital radiography to diagnose periapical lesions, especially when a working diagnosis is needed.
